# Correction: Singrang et al. Melatonin Inhibits Hypoxia-Induced Alzheimer’s Disease Pathogenesis by Regulating the Amyloidogenic Pathway in Human Neuroblastoma Cells. *Int. J. Mol. Sci.* 2024, *25*, 5225

**DOI:** 10.3390/ijms26157338

**Published:** 2025-07-30

**Authors:** Nongnuch Singrang, Chutikorn Nopparat, Jiraporn Panmanee, Piyarat Govitrapong

**Affiliations:** 1 Chulabhorn Graduate Institute, Bangkok 10210, Thailand; nuchsingh@gmail.com; 2 Innovative Learning Center, Srinakharinwirot University, Bangkok 10110, Thailand; m_chukorn@hotmail.com; 3 Research Center for Neuroscience, Institute of Molecular Biosciences, Mahidol University, Nakhon Pathom 73170, Thailand; jirapornpanmanee@gmail.com

## Error in Figure

In the original publication [1], there was a mistake in Figure 1a as published. The incorrect image was inadvertently used during final preparation. The corrected Figure 1a appears below.

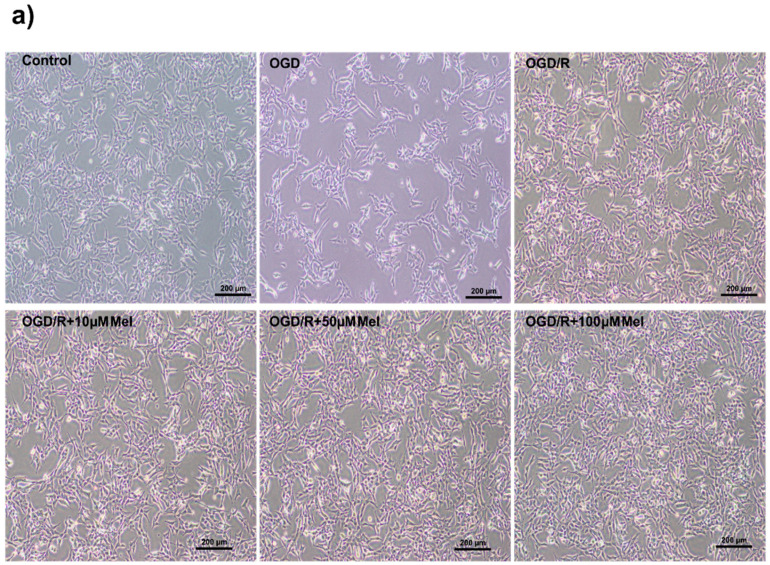



## Text Correction

There was an error in the original publication [1]: a correction has been made to the Methods section, in the subsection “Statistical Analysis”:

4.9. Statistical Analysis

For all the experiments, the data were analyzed, and statistical significance was determined using GraphPad Prism version 8.3.0. Differences between two groups were assessed using unpaired *t* tests (where equal variances were not assumed). Differences between multiple groups were assessed using a one-way analysis of variance (ANOVA), followed by Tukey’s multiple comparison test (where equal variances were not assumed). The data are expressed as the mean ± S.E.M. *p* values < 0.05 were considered to indicate statistical significance.

The authors state that the scientific conclusions are unaffected. This correction was approved by the Academic Editor. The original publication has also been updated.
